# Distal Transradial Access in the Anatomical Snuffbox for Interventional Coronary Procedures: Analysis of Access Site Pain and Complications

**DOI:** 10.7759/cureus.54878

**Published:** 2024-02-25

**Authors:** Roberto R Barbosa, Lucas De Barros, Rodolfo C Sylvestre, Vítor L Belloti, Guilherme F de Oliveira, Rodrigo D Ferraz, Bruno P de Aragão, Osmar A Calil, Renato Serpa, Luiz Fernando M Barbosa

**Affiliations:** 1 Cardiology, Hospital Santa Casa de Misericórdia de Vitória, Vitória, BRA; 2 Cardiology, Instituto Dante Pazzanese de Cardiologia, São Paulo, BRA; 3 Internal Medicine, Hospital das Clínicas da Faculdade de Medicina da Universidade de São Paulo (FMUSP), São Paulo, BRA

**Keywords:** cardiac catheterization, interventional cardiology, anatomical snuffbox, transfemoral access, distal transradial access, transradial access

## Abstract

Introduction: A novel arterial access distally on the radial artery through the anatomical snuffbox has been recently described for coronary interventional procedures. However, there is insufficient data comparing the advantages and limitations of distal transradial access (dTRA), conventional transradial access (TRA), and transfemoral access (TFA). The aim of this study was to compare the three access sites regarding local pain and complications during or after coronary interventional procedures.

Methods: This prospective observational single-center study included 211 patients undergoing cardiac catheterization or percutaneous coronary intervention, divided into three groups: dTRA (n=69), TRA (n=71), and TFA (n=71). The access site was chosen at the discretion of three operators. We administered a questionnaire to all patients, addressing local pain or discomfort during or after the procedure and the occurrence of possible complications such as distal pallor, local bleeding, and purple color on the access site.

Results: Pain on the access site during the procedure was reported more frequently in the TRA group (dTRA 15.9% vs. TRA 32.4% vs. TFA 15.5%). There were no differences in the occurrence of local pain after the procedure in all three groups (29.6% in the dTRA group, 28.2% in the TRA group, and 26.8% in the TFA group). Pain intensity, when it occurred, was higher in the dTRA group (dTRA 5.8 vs. TRA 4.8 vs. TFA 4.6 on a 1-10 scale), as was its duration (dTRA 13.7 vs. TRA 7.6 vs. TFA 8.2 days). Only two local bleeding events were reported, both in the TFA group. No major complications were recorded.

Conclusion: The occurrence of local pain on the puncture site after coronary interventional procedures did not differ among the three groups. The dTRA group presented a lower incidence of pain during the procedure when compared to TRA and a lower incidence of purple color when compared to TFA. However, pain intensity and duration were higher in the dTRA group when pain was reported. Using dTRA for coronary procedures is a feasible and safe strategy in selected cases.

## Introduction

Since 1989, after Campeau’s publication [1], transradial access (TRA) has been the focus of debate and has become an attractive technique for invasive coronary procedures. In present days, large studies sustain favorable results for TRA, such as low bleeding rates, fewer vascular complications, shorter hospitalization, and lower cost compared to transfemoral access (TFA) [1-5]. However, potential disadvantages, despite being uncommon, are still associated with the TRA approach, especially compartment syndrome, arterial occlusion, and arterial spasm [2-6]. Besides, some factors may influence the massive use of TRA, such as small radial arteries, abnormal Allen’s test, hemodialysis accesses on superior limbs, and unknown coronary artery bypass grafts after surgical myocardial revascularization [2].

A novel technique for TRA with a distal puncture in the anatomical snuffbox was described by Kiemeneji in 2017 [7]. This distal transradial access (dTRA) has certain advantages in comparison with traditional TRA once its puncture is made after the emergence of the superficial palmar arch, thus preserving the anterograde circulation by this arch and minimizing the risk of hand ischemia in cases of arterial occlusion [8]. Among other potential advantages, the neutral position of the patient’s arm, the reduced time for hemostasis, and the preservation of the radial artery for future hemodialysis fistulas should be cited [9].

Nonetheless, there are no large studies comparing dTRA with TRA and TFA regarding local pain and complications from these arterial accesses. The aim of this study was to compare the different access sites, analyze pain during or after the procedure, its intensity and duration, as well as puncture site complications.

## Materials and methods

Study design and population

This is a single-center prospective observational cohort study. Inclusion criteria were patients above 18 years old who underwent coronary angiography and/or percutaneous coronary intervention (PCI) between July 2019 and July 2020. Patients were consecutively included according to the access site in a non-randomized fashion until the completeness of each group (dTRA, TRA, and TFA). The initial access site was chosen at the discretion of one of three experienced operators, with no influence of the study on medical decisions. The indication for the procedure could be acute or chronic coronary syndromes. Patients were excluded if they were unconscious before or after the procedure, if they had a formal indication for oral anticoagulation, or if a right cardiac catheterization was performed. Another exclusion criterion was the crossover from one access site to another after an unsuccessful first attempt, since it might influence the perception of pain on different sites.

Data collection and analyzed variables

Patients were addressed right after the procedure, and those who accepted to participate signed the informed consent form. A questionnaire was applied to all included patients on the same day before discharge from the interventional cardiology department regarding the occurrence of pain during and after the procedure, pain intensity (on a 1-10 scale), local bleeding, purple color on the puncture site, or distal palloring. Then, within seven to 28 days, a follow-up visit was performed to assess possible access site complications and to reapply the questionnaire, now adding information about pain duration (in days). If the patient referred to ongoing pain at the time of the second questionnaire before day 28, the questionnaire would be reapplied on day 28.

Clinical characteristics such as age, gender, history of hypertension, diabetes mellitus, dyslipidemia, current smoking, chronic kidney disease, heart failure, previous peripheral artery disease, coronary artery disease, coronary artery bypass graft surgery, and the type of procedure (coronary angiography, PCI, or coronary angiography immediately followed by PCI) were collected for baseline analysis.

Procedures

The choice of access site was made at the discretion of at least one of the operators, according to individual preferences, experience, and technical possibilities. Three experienced operators performed the procedures that were included in the study. The determination of the access site was routinely done before the preparation of patients in the procedure room. dTRA punctures were performed in the anatomical snuffbox by pulse palpation of the radial artery; TRA punctures were made immediately above the styloid process; and TFA punctures were performed by pulse palpation of the femoral artery in the inguinal region. Ultrasound was not used in any case to guide puncture. Local anesthesia was used in all cases with infiltration of lidocaine 2% without vasoconstrictor (1 to 2 ml in dTRA and TRA punctures, 15 to 20 ml in TFA punctures). Conscious sedation was made on an individual basis, not routinely, at the discretion of the operators in each case, according to current local practices. Vascular sheaths and catheters were also chosen according to the operators’ preferences (sheath diameters 5, 6, or 7 French). Unfractionated heparin (UFH) was administered in all TRA and dTRA procedures (5,000 IU through the vascular sheath for coronary angiography and an additional intracoronary dose to reach 100 IU/kg for PCI). For TFA, weight-adjusted UFH was administered as an intracoronary bolus (100 IU/kg) if a PCI was performed, but no UFH was administered for coronary angiography. Only manual compression was performed for hemostasis in all access site groups.

Outcomes

The primary outcome of the study was local pain on the access site during or after the procedure. Secondary outcomes were pain intensity and duration and the occurrence of puncture site complications (active bleeding, purple color, or distal palloring as reported by patients in the questionnaires). If vascular complications were confirmed by medical staff, they would be counted as major complications, such as pseudoaneurysm, clinically important hematoma, limb ischemia, active bleeding at the insertion site, retroperitoneal hemorrhage, compartment syndrome, or arteriovenous fistula formation.

Possible minor complications were questioned in a dichotomic fashion with yes-or-no questions. Active bleeding and purple color were considered surrogates for hematomas or bruises, and distal palloring for possible limb ischemia caused by arterial occlusion. Patients were encouraged to return for medical evaluation if there was any suspicion of complications, regardless of scheduling a medical appointment.

Statistical analysis

A sample size calculation of 210 patients was made, matching the three groups with 70 patients in each, predicting a statistically significant difference among groups for the outcome of “pain during the procedure." The estimates used for the calculation were 40% in groups dTRA and TRA and 20% in group TFA, with an 80% power. Statistical analysis comprehended the chi-square test, the Fisher exact test, and the unpaired t-test, with a 5% level of significance, using SPSS Statistics version 26.0 (IBM Corp. Released 2019. IBM SPSS Statistics for Windows, Version 26.0. Armonk, NY: IBM Corp).

Ethical aspects

This study was conducted in accordance with current ethical standards, in agreement with the Declaration of Helsinki and Resolution 466/2012 from the Brazilian Ministry of Health. The study project had been previously approved by the Research Ethics Committee of Santa Casa de Misericórdia de Vitória School of Sciences (approval number: 3.447.088). An informed consent term was applied to and signed by all included patients in this study.

## Results

Initially, 212 patients were included. One patient was excluded due to a crossover of the access site (from dTRA to TRA), resulting in 211 patients that composed the final sample, divided into groups dTRA (n=69), TRA (n=71), and TFA (n=71). The mean age was 64.5 ± 9.7 years; the male gender comprised 126 (59.7%) and the female gender comprised 85 (40.3%) of the patients. The prevalence of hypertension in the overall population was 77.2% (163 patients), and diabetes was 38.4% (81 patients). Coronary angiography was performed in 62.1% (131 patients), PCI in 25.6% (54 patients), and coronary angiography was followed by PCI in 12.3% (26 patients) of all patients.

There was a higher prevalence of male gender in group dTRA when compared to TRA. Group TFA had a higher prevalence of chronic kidney failure, heart failure, and a previous history of coronary artery disease compared to group TRA. Previous coronary artery bypass graft surgery was more common in group TFA compared to both other groups. Data referring to baseline clinical characteristics are expressed in Table 1.

**Table 1:** Baseline clinical characteristics of dTRA, TRA, and TFA groups dTRA: distal transradial access, TRA: transradial access, TFA: transfemoral access, CAD: coronary artery disease, PAD: peripheral artery disease, PCI: percutaneous coronary intervention, CABG: coronary artery bypass graft, P1: p-value comparing groups dTRA and TRA, P2: p-value comparing groups dTRA and TFA, P3: p-value comparing groups TRA and TFA

Clinical variable	dTRA	TRA	TFA	P1 / P2 / P3
Age (years), average ± SD	64.0 ± 10,2	64.3 ± 9.9	65.1 ± 9.1	0.51 / 0.74 / 0.69
Male gender, n (%)	49 (69.0%)	37 (52.1%)	40 (56.3%)	0.021 / 0.07 / 0.61
Female gender, n (%)	20 (28.2%)	34 (47.9%)	31 (43.7%)	0.021 / 0.07 / 0.61
Hypertension, n (%)	48 (67.6%)	56 (78.9%)	59 (83.1%)	0.21 / 0.059 / 0.52
Diabetes mellitus, n (%)	26 (36.6%)	27 (38.0%)	28 (39.4%)	1 / 0.83 / 0.86
Dyslipidemia, n (%)	40 (56.3%)	43 (60.6%)	45 (63.4%)	0.75 / 0.51 / 0.72
Current smoking, n (%)	12 (16.9%)	8 (11.3%)	9 (12.7%)	0.3 / 0.43 / 0.79
Chronic kidney disease, n (%)	4 (5.6%)	7 (9.9%)	15 (21.1%)	0.35 / 0.008 / 0.06
Serum creatinine, average ± SD	1.01 ± 0.26	1.05 ± 0.5	1.30 ± 1.1	0.55 / 0.034 / 0.085
Previous PAD, n (%)	2 (2.8%)	2 (2.8%)	1 (1.4%)	0.97 / 0.54 / 0.55
Previous CAD, n (%)	17 (23.9%)	20 (28.2%)	30 (42.3%)	0.63 / 0.02 / 0.07
Previous PCI, n (%)	12 (16.9%)	15 (21.1%)	17 (23.9%)	0.57 / 0.33 / 0.68
Previous CABG surgery, n (%)	3 (4.4%)	3 (4.2%)	18 (25.4%)	0.97 / 0.0005 / 0.0004
Heart failure, n (%)	7 (9.9%)	11 (15.5%)	16 (22.5%)	0.34 / 0.047 / 0.28

Patients who underwent coronary angiography followed by PCI presented a higher use of dTRA and TFA than TRA. The frequency of patients who underwent coronary angiography only or PCI only did not differ significantly among groups, as shown in Table 2.

**Table 2:** Type of procedure performed among dTRA, TRA, and TFA groups dTRA: distal transradial access, TRA: transradial access, TFA: transfemoral access, PCI: percutaneous coronary intervention, P1: p-value comparing groups dTRA and TRA, P2: p-value comparing groups dTRA and TFA, P3: p-value comparing groups TRA and TFA

Type of procedure	dTRA	TRA	TFA	P1 / P2 / P3
Coronary angiography, n (%)	39 (54.9%)	51 (71.8%)	41 (57.7%)	0.058 / 0.88 / 0.078
PCI, n (%)	17 (23.9%)	18 (25.4%)	19 (26.8%)	1 / 0.77 / 0.84
Coronary angiography followed by PCI, n (%)	13 (18.3%)	2 (2.8%)	11 (15.5%)	0.002 / 0.59 / 0.008

The median time from the procedure until the application of the second questionnaire was nine days. Local pain during the procedure was more frequent in group TRA compared to groups dTRA and TFA. The occurrence of pain after the procedure was similar among groups (dTRA, TRA, and TFA). The intensity and duration of local pain, when it occurred, were higher in group dTRA. The comparison of pain during and after the procedure among the three groups is described in Table 3.

**Table 3:** Analysis of pain on access site among dTRA, TRA, and TFA groups dTRA: distal transradial access, TRA: transradial access, TFA: transfemoral access, P1: p-value comparing groups dTRA and TRA, P2: p-value comparing groups dTRA and TFA, P3: p-value comparing groups TRA and TFA

Characteristics of pain	dTRA	TRA	TFA	P1 / P2 / P3
Pain during procedure, n (%)	11 (15.9%)	23 (32.4%)	11 (15.5%)	0.023 / 1 / 0.018
Pain after procedure, n (%)	21 (29.6%)	20 (28.2%)	19 (26.8%)	0.76 / 0.63 / 0.85
Pain intensity (1 to 10), average ± SD	5.8 ± 2.2	4.8 ± 2.5	4.6 ± 1.7	0.013 / 0.0004 / 0.57
Pain duration (days), average ± SD	13.7 ± 9.8	7.6 ± 5.2	8.2 ± 4.1	0.0001 / 0.0001 / 0.44

Regarding possible minor vascular complications, the occurrence of purple color was higher in the TFA group compared to the dTRA, while distal palloring was more frequent in the TRA group compared to the dTRA. In fact, the dTRA group did not present any reports of distal palloring. No major vascular complications were diagnosed by medical staff in the total sample, not even in patients who reported alterations in the questionnaire. These self-reported minor complications are shown in Table 4.

**Table 4:** Analysis of possible minor complications according to the questionnaire among dTRA, TRA, and TFA groups dTRA: distal transradial access, TRA: transradial access, TFA: transfemoral access, P1: p-value comparing groups dTRA and TRA, P2: p-value comparing groups dTRA and TFA, P3: p-value comparing groups TRA and TFA

Complications	dTRA	TRA	TFA	P1 / P2 / P3
Purplish color, n (%)	15 (21.1%)	24 (33.8%)	28 (39.4%)	0.11 / 0.023 / 0.48
Distal palloring, n (%)	0	3 (4.2%)	1 (1.4%)	0.034 / 0.22 / 0.31
Bleeding, n (%)	0	0	2 (2.8%)	1 / 0.08 / 0.08

## Discussion

Our study analyzed local pain and alterations on the puncture site that were self-reported. Pain after the procedure and active bleeding were similar among the dTRA, TRA, and TFA groups, although pain during the procedure was more common in the TRA group. However, when pain was reported, the dTRA group presented higher intensity and duration. Besides, the dTRA group had a lower incidence of self-reported complications - purple color and distal palloring - compared to the TFA and TRA groups, respectively.

The transradial approach has multiple advantages over the femoral approach for coronary procedures. Besides early deambulation, shorter hospitalization, and greater comfort for patients during the procedure [3,10-12], there is a lower risk of death from all causes (OR = 0.71), major bleeding events (OR = 0.53), and major cardiovascular events (OR = 0.84) when using TRA [13]. These reductions are even more significant in the context of acute coronary syndromes, in which the aggressive use of antiplatelets and antithrombotic agents may increase the risk of bleeding events, especially those associated with femoral artery access [14]. Thus, the TRA is recommended by guidelines and is preferable in cases of ST-elevation myocardial infarction for the prevention of vascular bleeding complications [15-18].

Recently, a novel transradial technique was described by puncturing the radial artery distally through the anatomical snuffbox after the emergence of the palmar arch [19]. The dTRA has potential advantages over the conventional TRA, such as the neutral position of the patient’s arm, equally low or lower rates of arterial occlusion, shorter hemostasis time, and a lower risk of compartment syndrome and hematoma [9,20]. Although it may be challenging at first, the dTRA has been used by many interventional cardiologists, mainly those experienced with transradial and other forearm techniques.

Koutouzis et al. analyzed patients’ satisfaction regarding the access site, and no difference was observed between groups dTRA and TRA [21]. Respectively, 79% and 85% of patients in each group answered positively about using the same access site for future procedures. However, this study differs from ours by questioning only the willingness to use the same puncture site if a new cardiac catheterization is required in the future. Other studies on this subject did not directly address local pain as ours did and contained only subjective questions in satisfaction questionnaires [8,22].

A few disadvantages of the dTRA have been reported, such as a longer learning curve for operators, which causes a higher crossover rate and multiple punctures, besides a longer duration of procedures [9]. Some have found difficulties in puncturing the radial artery in the anatomical snuffbox due to its lower diameter, deeper location, and tortuosity [6,7,9,23]. Numerous attempts may cause pain, and this could explain the pain intensity and duration, which were higher in the dTRA group. However, only one case of access site failure and crossover occurred in our study. These results confirm that dTRA is a new possibility for coronary catheterization and a great option that should be chosen on an individual basis.

In the past few years, a number of studies have demonstrated high success rates for dTRA in interventional cardiology, ranging from 88% to 100% in most of them [9,10,15,21]. In discordance, one randomized study demonstrated an inferior result (70% success rate) [21]. Our study presented a high success rate for the dTRA group (98.6%). Since the access site crossover was an exclusion criterion, local pain or purple color in this single case was not assessed. The non-randomized nature of our study may have led to a higher success rate of dTRA if compared to real-world patients, where lower rates may be achieved, especially in women, the elderly, and cardiogenic shock patients.

The explanation for similar incidences of pain during the procedure in all three groups could reside in the experience of the operators with different access sites. Besides, access to site selection prior to procedure preparation and, according to individual feasibility, supports this finding. Once pain after the procedure was more common in the TRA group, this could be explained by local characteristics rather than by the procedure itself, i.e., excessive hand movements and compression of the puncture site when the hand is deflected. Another possible reason is the higher rate of spasm of the radial artery next to the styloid process or even symptomatic occlusions of the radial artery at this location. However, these reasons were not properly addressed by our study, and the routine use of ultrasonography would be able to answer some of these questions. Fortunately, despite the small sample size, no major complications at puncture sites were observed.

Although relevant, our study has some limitations. Firstly, it is observational in nature, which impairs the extrapolation of results. Some procedural features were not assessed and were heterogeneous: vascular sheath diameters, use of sedative agents, and number of puncture attempts, which could lead to an important bias regarding pain or discomfort. Secondly, analyzing ischemia or bleeding events using surrogate endpoints that are self-reported may overestimate possible minor complications since patients tend to value symptoms when actively questioned. Also, radial artery patency was not evaluated by ultrasound, which should be mandatory for confirming this outcome, even though it is frequently asymptomatic. Yet, to our knowledge, our study is the first to directly compare the three most common access sites for cardiac catheterization regarding pain during and after the procedure.

## Conclusions

The dTRA group presented a lower incidence of local pain during the procedure compared to the TRA group and a lower incidence of local purple color compared to the TFA group. There were no significant differences in the occurrence of pain after the procedure, distal palloring, or local bleeding among the three groups. When pain was reported (less than one-third of patients in each group), the dTRA group had higher pain intensity and duration than the TRA and TFA groups. No major complications related to arterial access sites occurred. These findings confirm the dTRA as a feasible, safe, and comfortable access site choice, making it an attractive option for arterial access in interventional cardiology in appropriate cases.
